# First comprehensive contribution to medical ethnobotany of Western Pyrenees

**DOI:** 10.1186/1746-4269-3-26

**Published:** 2007-06-06

**Authors:** Silvia Akerreta, Rita Yolanda Cavero, María Isabel Calvo

**Affiliations:** 1 Department of Plant Biology (Botany Section), University of Navarra, C/Irunlarrea s/n, Pamplona, 31080, Navarra, Spain; 2 Department of Pharmacy and Pharmaceutical Technology (Pharmacognosy Section), University of Navarra, C/Irunlarrea s/n, Pamplona. 31080, Navarra, Spain

## Abstract

**Background:**

An ethnobotanical and medical study was carried out in the Navarre Pyrenees, an area known both for its high biological diversity and its cultural significance.

As well as the compilation of an ethnopharmacological catalogue, a quantitative ethnobotanical comparison has been carried out in relation to the outcomes from other studies about the Pyrenees. A review of all drugs used in the area has also been carried out, through a study of the monographs published by the institutions and organizations responsible for the safety and efficacy of medicinal plants (WHO, ESCOP, and the E Commission of the German Department of Health) in order to ascertain the extent to which the Navarre Pyrenees ethnopharmacology has been officially evaluated.

**Methods:**

Fieldwork was carried out over two years, from November 2004 to December 2006. During that time we interviewed 88 local people in 40 villages. Information was collected using semi-structured ethnobotanical interviews and the data was analyzed using quantitave indexes: Ethnobotonicity Index, Shannon-Wiener's Diversity, Equitability and The Informant Consensus Factor. The official review has been performed using the official monographs published by the WHO, ESCOP and the E Commission of the German Department of Health.

**Results:**

The ethnobotanical and medical catalogue of the Navarre Pyrenees Area comprises 92 species, of which 39 have been mentioned by at least three interviewees. The quantitative ethnobotany results show lower values than those found in other studies about the Pyrenees; and 57.6% of the Pyrenees medical ethnobotany described does not figure in documents published by the above mentioned institutions.

**Conclusion:**

The results show a reduction in the ethnobotanical and medical knowledge in the area of study, when compared to other studies carried out in the Pyrenees. Nevertheless, the use of several species that may be regarded as possible sources for pharmacological studies is reported here such as the bark of *Sambucus nigra*, the roots of *Fragaria vesca*, or the leaves of *Scrophularia nodosa*. These species are not currently approved by the WHO, ESCOP and the E Commission of the German Department of Health, institutions that, apart from encouraging the greater use of plants for medicinal purposes, may help in the design of development plans for these rural areas by validating their traditional medicine.

## 1. Background

Ethnobotany is the study of the utilitarian relationship between human beings and vegetation in their environment, including medicinal uses [1]. Its importance lies in the fact that in addition to contributing to knowledge and conservation of features of ancestral popular culture, it opens up the possibility of finding new uses for medicinal plants, and can serve to discover new medicines derived from plants [2].

For this study, an interdisciplinary research team was set up, between the Department of Plant Biology (Botany) and the Department of Pharmacy and Pharmaceutical Technology (Pharmacognosy) of the University of Navarra, Spain. The project was started in 2003 and it was intended to reverse the trend towards fragmentation in research groups [3,4], in order to achieve a deeper knowledge of the plants used in Navarre's traditional medicine, their active principles, composition and therapeutic use; this is the context in which the research project concerning the Pyrenean region has been carried out.

The Pyrenees are a mountain range, of 435 km in length from east to west, which divides the Iberian Peninsula from the rest of Europe. It presents a great variety of climates and soils, which in turn produce a rich ecology and flora (3500 taxa in total [5], an important figure when read in relation to the 7500 species that have been catalogued in the Iberian Peninsula and the Balearic Islands as a whole, among the highest rates in Europe [6]). It is a relatively small territory which has nonetheless seen the survival of six different languages from different cultural traditions and has attracted the attention of many explorers and botanists throughout the centuries. After touring its landscapes and villages, these explorers and botanists reported their findings [7,8]. However, ethnobotanical studies began in earnest about two decades ago, when research groups in this field were first established on the Iberian Peninsula [9-40]. In fact, ethnobotanical research in the Pyrenees has been carried out only in some regions of the Eastern and Central areas [6,41-49]; with regard to the Western area, only a few divulgative publications about medicinal flora or edible plants have appeared, but these studies were not produced using an ethnobotanical methodology [50-55].

### Study area

**Figure 1:**
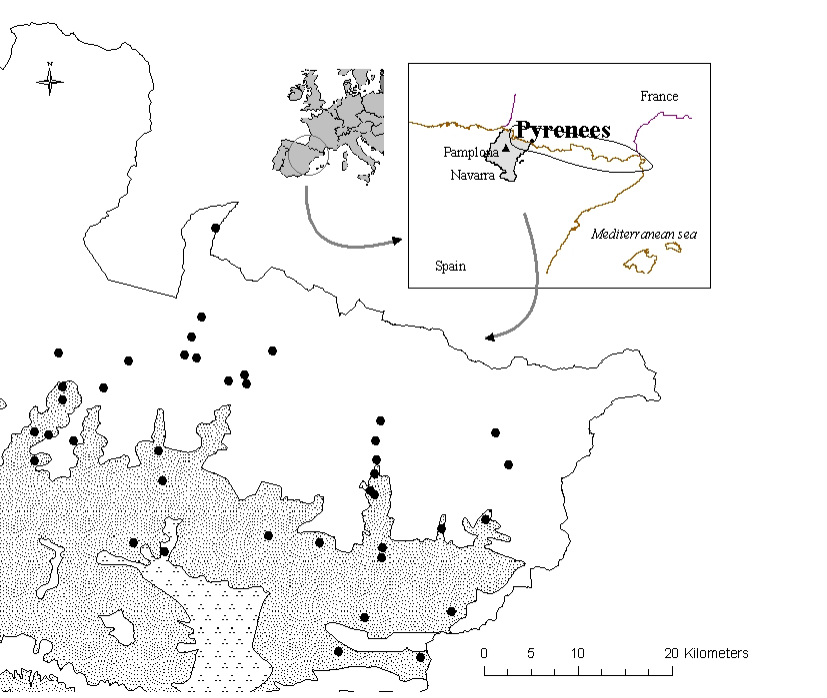
**Location of the area studied in Europe and in Navarra**. **• **Visited villages **Bioclimates **Blank area: Temperate oceanic Random dotted area: Temperate oceanic sub-mediterranean Aggregately dotted area: Mediterranean pluviseasonal.

The Navarre Pyrenees area, approximately 2200 km^2^, is situated in the north-east of Navarra and in the south-western part of the Pyrenees (Figure 1). It is a mountainous region with acute unevenness of terrain (from 245 metres over sea-level to 2428 metres at the summit of its highest mountain). The area has a mild oceanic climate in its northern part which shifts to a submediterranean climate type as one moves to the southern areas of Navarre [56]; this fact makes it different from other Pyrenean regions (Central and Eastern Pyrenees), which exhibit a basically Mediterranean climate [57]. Lithology is varied: limestone mountains dating from the Upper Cretaceous Era rise out of valleys which are composed mainly of Palaeozoic and Eocene flysch (Tertiary Era), with marls and limestone marls from the Mid and Upper Eocene Era towards the western fringe [58].

All this produces a distinctive flora consisting of about 1822 taxa [7], including the species of the adjacent Pyrenean regions. Forests such as those of *Fagus sylvatica *L. and of *Quercus humilis *L. are plentiful, as well as of *Pinus sylvestris *L., *Acer opalus *Miller, *Buxus sempervirens *L., *Lonicera etrusca *G. Santi, *Viburnum lantana *L., of various species of the *Rosa *genus [59], and of endemic species such as *Campanula ficarioides *Timb.-Lagr. ssp. *orhyi*, *Alchemilla iratiana *S.E Fröhner or *Euphorbia pyrenaica *Jord, among others. [7].

Given its biological and cultural richness, the region currently enjoys a good variety of protected areas: 3 integral reservation zones, 8 natural reservation zones, 14 locations proposed as Sites of Community Interest (SCIs) within the Natura network, and 11 flower areas introduced by Blanco [60] in his areas of botanical interest (such as the Irati forest, one of the most important beech woods in Europe), along with a further 31 zones under diverse forms of biological protection.

In the seven valleys of the Navarre Pyrenees human settlement dates back 7,000 years, as has been proven by archaeological findings in the Aizpea Cave [61]. The first inhabitants in the region, the Vascones, were influenced by several cultures that sought to settle in this territory as well, such as the Romans and the Visigoths. Nowadays, the population in this area lives in small villages and houses scattered across the hills and valleys. They earn a living through cattle raising, hand-made exploitation of forests and the cultivation of a variety of vegetables for consumption. Yet, these activities have been reduced in the shift towards computer and service industries and because of emigration to large centres of population. Despite the fact that cultural traditions have remained well-rooted in the region, only 10% of the population speaks 'Euskara', the language of the Basque ethnic group; the percentage of Euskara speakers was higher at other times in recent history (18^th ^and 19^th ^centuries) [50], a fact which influences different aspects of the present culture, including ethnobotany.

All these biotic and cultural factors, and the need for a scientific review of the pharmacology of the Navarre Pyrenees, constitute the primary motive of this study, whose objectives are:

-to compile an ethnobotanical and medical catalogue of the Navarre Pyrenean Region;

-to perform a quantitative analysis of the results and to compare it with other ethnobotanical studies of the Pyrenees region;

-to carry out a review of the drugs used within the community through study of the monographs published by WHO, ESCOP and the E Commission of the German Department of Health, institutions responsible for the safety and efficacy of medicinal plants, in order to assess the official validity of the pharmacology of the Navarre Pyrenees.

## 2. Material and methods

Field work was carried out over two years, from November 2004 to December 2006. The methodology used to elicit information was ethnobotanical interviews with people with a knowledge of traditional medicine. Many villages and towns have been visited and many of their local people have helped in locating of the interviewees and making first contact. Interviews were individual, except in some cases where several people participated at the same time, as happened in an interview carried out in a nursing home. Interviews did not follow a previously-defined strict questionnaire [10]; they consisted of open [62] and semi-structured [63] questions, which allow and encourage the interviewees' spontaneous participation. The languages used with informants was Spanish and, in some cases, Basque. Whenever possible, the conversations were recorded with the consent of the interviewees and more than one interview session was carried out with some participants to confirm the identification of some plants.

88 people from 40 different villages and towns were interviewed, all of them local people without scientific knowledge, born or having lived most of their lives in the region. 55,7% were women, with ages ranging from 31 to 100 years (average: 68). The species were collected with the interviewee *in situ *and identified in the laboratory using keys for botanical determination and labelled and included in the PAMP Herbarium at the School of Sciences. The bibliography used for taxonomic determination was *Flora Iberica *[64], and for unpublished families, we have used *Flora of the Basque Country *[65].

The plants and their uses were subsequently catalogued according to therapeutic application. For this purpose, a set of 12 categories was established (Table 1), based on the catalogue of pharmaceutical specialities [66] and the traditional terms for illness used in the region, which have two origins: one in which there is a natural explanation for illness; and the other, more spiritual or supernatural, in which illness is caused by the evil eye or magic, but may be treated or cured in the same way [67]. Thus, one of the categories includes 'other traditional uses', where this second type of illness is under discussion. Once the information had been processed, the ethnobotanical information collected was also analyzed quantitatively to obtain the following data: most cited species, plants to which most uses were attributed, number of different uses and most reported medicinal uses, parts of plants most frequently mentioned and the most commonly mentioned preparation and application processes. To establish a deeper pharmacological knowledge of the Navarre Pyrenees, the data was also assessed using quantitative analyses:

**Table 1:** List of Pharmaceutic Categories.

**Number**	**Category**	**Main therapeutic applications**
1	Cardiovascular system and haematology	Hypertension, cardiovascular diseases...
2	Dermatology	Warts, wounds, spots, burns...
3	Endocrinology, reproductive system, liver and gall bladder	Menstrual pain, reproductive system disorders....
4	Gastrointestinal tract	Stomach ache, diarrhoea...
5	Joints and rheumatism	Rheumatism, tendons and joints troubles, osteoarthritis, muscular pains...
6	Infectious diseases, fever, anti-inflammatory and immunostimulating treatment	Infectious diseases, fever, anti-inflammatory and immunostimulating treatment
7	Neurology and psychiatry	Tranquillizer, stimulating...
8	Respiratory tract	Cold, bronchitis, asthma...
9	Urinary tract	Diuretic, depurative, prostate troubles, renal calculus...
10	Ears, nose, throat, mouth and ophthalmology	Sore throat, earache, eyes problems, toothaches,...
11	Other traditional applications	Foot odour, 'old diseases' and other advice to bring health and good luck.
12	Veterinary uses	Animal disorders

1) the Portéres' Ethnobotanical Index [44], which consists in the ratio between useful plants and total flora expressed as a percentage, and which has been adapted to medicinal plants in order to obtain a pharmacological ethnobotanical index [45], so that it can be compared to the results obtained by researchers in the eastern Pyrenees region;

2) ethnobotanical diversity assessed through the Shannon-Wiener index, H' = -Σ (p_i _ln p_i_), where p_i _is the proportion of the number of citations for each species relative to the total number of citations [68];

3) equitability E = H'/H_max _(where H_max _= in R, and R = number of medicinal plants); and

4) FIC, informant consensus factor, the ratio between the number of use-reports minus the number of medicinal plants and the number of use reports minus one [45].

Finally, the uses of every plant have been checked against information published by WHO [69], ESCOP [70] and the E Commission of the German Department of Health [71]. The monographs published by these institutions were reviewed for every organ or part of the reported plants, and the percentage was calculated by dividing the number of plants on which monographs have been published (both positive or negative) into the total number of medicinal plants used.

## 3. Results and discussion

### Synthesis of global results

Botanical remedies of the folk pharmacopoeia in the Pyrenean region of Navarra, comprise 92 species (see additional file 1). Of these species, 39 species were cited by three or more informants. This does not mean that a cure or species mentioned by only one or two informants is without value, but may simply reflect the disappearance of particular knowledge; the fact of being reported on a relatively low scale may also mean that these plants are worth of further study.

### Botanical analyses: families, species and parts of the plants

The predominating botanical families are *Asteraceae *(15 species), *Lamiaceae *(13 species) and *Rosaceae *(8 species). Only a few botanical families mentioned by the informants comprise between one-third and one-half of the total number of plants cited. This corresponds to other ethnobotanical studies carried out using the same method in the Pyrenees [44]. It is not surprising, because these families are well represented in the Pyrenees flora and contain some very common plants (the *Asteraceae *family is the most important of them, and *Lamiaceae *and *Rosaceae *are among the top seven) [7]. As confirmed by Johns *et al*. [72] and other authors [18,41,44], the more common a plant is in an area, the greater the probability of its popular use.

*Sambucus nigra *is the most reported species and almost all its organs are used and many of their applications are also mentioned in Bonet *et al*. [44], Agelet and Vallès [42] and Vallès *et al*. [15]. Apart from the fact that different parts of the plant are used, harvesting of the plant, practically all year round, has been reported, which encourages its continued use generation after generation. Other plants in significant use, as recorded in other studies about the Pyrenees, are: *Santolina chamaecyparissus *ssp. *squarrosa *and *Urtica dioica *(stinging nettle).

The parts of the plants most used for medicinal purposes are, in descending order: leaves, flowers (including floral summits and flowering heads), aerial parts and fruits, results similar to those observed by Bonet *et al*. [44] and very similar to those obtained by Cerdanya [45,46]. According to Bonet *et al*. [44], the reason why leaves are used most is because they are easily accessible. Besides, recalling the line of reasoning proposed by Johns *et al*. did is remember ('the more common a plant in the area, the higher its probability of use'), if the leaf is the most accessible or common part in the flora of the area, and the most abundant as well, it is more likely to be used. It was also observed that the interviewees showed a marked inclination to using leaves in the process of identifying and distinguishing medicinal plants. Thus, if the leaf is a key factor in the identification of plants, and of frequent and easy access, it follows that it would be more used than other plant organs.

### Preparation and administration

The percentage of external uses (54.4%) is lightly higher than internal uses (45.6%) and infusion is the main method of preparation for oral administration, as reported in the majority of the Pyrenean studies [41,44]. It is also important to point out that it is not always easy to differentiate this procedure from decoction on the basis of information given in interviews, a fact confirmed by several authors [9,45,73].

Regarding external uses, the most common is the poultice, according to the data collected in this study and in others on the central and eastern Pyrenees. [41,44,48]. In the Navarre Pyrenees, there were 20 references mentioned maceration as a method of preparation. The most usual is maceration with anisette (with a use entirely internal and digestive), as in the case of the preparate called 'patxaran', the most famous and characteristic liquor not only of the Pyrenean region but also of Navarra as a whole. Its production and consumption has also been reported in certain neighbouring villages and towns in the central Pyrenees [48]. Other liquors with digestive uses are produced; for example "patxaka" are produced, which is prepared from anisette and rosehips from several species of the genus *Rosa*. Maceration in alcohol (always used externally), oil or anisette and its traditional use have been preserved due to the easy process of preparation and the long durability of the products.

Only a few medicinal species are used as edible plants. This fact is widely different to the rest of Pyrenees regions. The explanation is that the number of medicinal food plants from the rest of the Pyrenees is similar to other European Mediterranean areas, whereas our data alike other Temperate areas from northern Iberian Peninsula [16,20]. In addition the informants clearly distinguish between culinary and medicinal uses alike other Temperate areas, although these use may sometimes overlap [20,21].

Another important issue to be pointed out is the fact that whereas in other studies about the Pyrenees, the use of liquors or teas has been regarded as falling between medicinal and edible uses because of its social character [44], this is not the case in this study because, although the use of some liquors has been socialized, these liquors continue to be consumed for medicinal purposes.

Almost all of the species are used alone, very few mixtures have been identified, such as the use of garlic and verbena in the production of poultices, or the use of garlic and elder in the preparation of ointments. The widespread use of garlic, mainly in dermatological preparations, shows the popular valued attributed to this species, although interviewees pointed out that the plants accompanying the garlic usually perform the most significant role.

In Navarre Pyrenees the number nine is repeated in methods concerning the popular administration of plants, which are always given for nine consecutive days (known as a "novena") during the seasons of autumn and spring, as also happens in other cultures in the Pyrenees [41,42,44]. From the ethnographic point of view this phenomenon reflects the idea that the number nine represents a magical number in traditional medicine [77,78] or, as Bonet *et al*. [44] indicate, the idea that the use of the plant over more days could have harmful effects for the organism.

The use of medicinal plants in certain symbolic rites still occurs in the Navarre Pyrenees. The informants do not positively state whether the healing principle is the rite or the plant. Nevertheless, as happens in other places in the Pyrenees, people still place certain previously blessed species [48] on the doors of the houses, to protect them from illness. This has no scientific basis nowadays, but folklore continues to give equal importance to both conceptions of illness and remedy.

### Common local plant names

Informants used 112 local names to refer to the 92 medicinal species catalogued, 14 of them in Basque (12,5%), a slightly higher percentage than that of Basque-speakers (10%). Among the Spanish names 10 refer to the place where the plants are recollected ("manzanilla de monte", mountain camomile, "té de roca", rock tea); others refer to their attributed use ("hierba para las almorranas", herb for piles, because of its use on haemorrhoids, "hierba para las piedras del riñon" herb for kidney stones); or its resemblance to animals ("patamula", mule's leg; reported as well in the neighbouring Pyrenean region [48], "pedo de lobo" wolf's fart or "cola de caballo" horse's tail). Special mention should be made of "cabardera" or "cabarda" to name bushes from the genus *Rosa*, most likely with the same phytonomous origin as that of the "gabarderas" reported in the ethnobotanical study of the Central Pyrenees [48]. These references have also been found among the Basque names recorded, such as plant names based on their use "pasmobelarra", amazement herb, or "iodobelarra", iodine herb, because of its resemblance to this chemical product. Mistletoe is commonly known in the area as "bizko", "migula", "mihura" (from Basque "mi", a corruption of "mamia", flesh or pulp, and "ura", water, which refers to the fruit [79], or the name "patxaran", which comes from the word "basarana" "basoa", forest, and "arana" plum: forest plum. A name related to the sun, "eguzkilore", or sun thistle [80], has been reported as well, which reflects the close relationship that ancient dwellers had with their environment and is used to protect against "evil spirits" and illnesses at night.

### Drug functions

A total of 200 popular uses have been compiled, in which dermatology is the most frequently cited category, followed by those categories related to gastrointestinal problems and the respiratory tract. In other regions of the Pyrenees more or less the same uses have been recorded, digestive and dermatological categories being likewise the most important ones [41,44,46,48].

This is not surprising, given the fact that, as Bonet and Muntané mention [44,45], the way work and life is led in rural areas and the lack of health and hygienic conditions, encouraged the search for natural remedies to cure infected wounds caused in daily life, or tisanes that helped the digestion of high calorie meals eaten to withstand cold temperatures. It should be borne in mind that most of the informants lived an exclusively rural way of life until approximately two decades ago.

Another point of interest is that traditional cures are usually limited to the treatment of mild and chronic diseases, such as Reuter and Bonet studied in other regions [mentioned in 44]. However, it is known that quacks from the Navarre Pyrenees usually treated serious diseases with medicinal plants when people were not able to treat them with the remedies that are presented above. Although those quacks have been reported in many European ethnobotanical research studies [81,82], it is not possible to find them on Navarre Pyrenees as they died.

### Data on quantitative ethnobotany

Table 2 shows the results of some quantitative data from the Navarra Pyrenees, as well as the results of other studies performed in the Pyrenees. The radius MP/Km^2 ^is slightly higher than that of the neighbouring Pyrenean region [mentioned in 48], but clearly lower than some results from the eastern Pyrenees, with a more Mediterranean character, which may have a strong influence on the presence and use of more species in a region milder than the Navarre Pyrenees.

**Table 2:** Comparison of results of ethnobotanical studies of various Pyrenean regions.

Region [^a^]	Location^b^	Extension (km^2^)	Population	Flora	MP^c^	MP/km^2^	NI^d^	MP/I^e^	EI^f^
Pallars [41-43]	E	2530	18800	1500	437	0.17	264	1.7	29.1
Cerdanya [46]	E	1086	23000	1600	234	0.21	155	1.5	15
Alt Empordà [40,44]	E	178	41300	1650	300	1.68	195	1.53	18
Cerdanya i Conflent [45]	E	2200	45000	-	225	0.10	208	1.03	-
Pirineo oscense [5,48]	C	15671	222000	2656	553	0.035	-	-	20.82
Pirineo navarro*	W	2200	7800	1822	92	0.042	88	1.04	5.05

^a ^Reference

^b ^Location in Pyrenees: C: central Pyrenees; E: eastern Pyrenees; W: western Pyrenees.

^c ^Number of medicinal plants cited

^d ^Number of informants

^e ^Number of medicinal plants cited per informant

^f ^Ethnobotanicity index

* Present study

The pharmacological ethnobotany index (EI) is clearly lower (5,05%) than the other with which this one has been compared, which suggests:

a) from the floral data of an area larger (the Pyrenees and the Prepyrenees) than that of this study (only the Pyrenees), the resulting EI is affected and shows a lower value than the actual one (EI = 5,05);

b) it is possible that the data reflects a cultural loss in the ethnobotanical and medical knowledge in the area, as suggested by the relatively low number of species (39) reported by at least three of the informants;

c) according to Mesa-Jiménez [83], a smaller number of medicinal plants used by a community means a higher rate of validation of those plants, because their efficacy has meant that other remedies have not been sought among other species, and therefore shows a greater level of adaptation of the inhabitants to their environment. In order to check if this study reflects this theory, the Shannon-Wiener index and the Equitability Index have been calculated. H' = 3,855, which means high diversity because the maximum value (H_max_) is 4,521; and E = 0,85 (value close to the maximum, which is 1). Therefore, these indexes show that the level of adaptation to the environment is low, according to Mesa-Jimenez's argument.

Besides, the FIC value is 0,65. The value of this index (ranging from 0 to 1) for the area studied, despite being high, is considerably lower than values calculated in several areas of the Iberian Peninsula: 0,85 and 0,91 for a Portuguese and a Catalan region respectively [36,14].

### Relation between traditional pharmacopeia and international organization

For each therapeutic category established, Table 3 shows the comparison between the popular use of drugs and their evaluation by the WHO, ESCOP and the E Commission, as well as the references to each plant in relation to each therapeutic application. In this table, taxa are ordered alphabetically and there are missing species that is, species with no monograph history.

**Table 3:** Medicinal plants with Monograph (revision on WHO, ESCOP and Commission E)

**Therapeutic category **^**a**^	1			2			3			4			5			6			7			8			9			10			11			12
	
**Species Bibliography **^**b**^	E	S	W	E	S	W	E	S	W	E	S	W	E	S	W	E	S	W	E	S	W	E	S	W	E	S	W	E	S	W	E	S	W	
*Achillea millefolium*				■																														
*Agrimonia eupatoria*																						■												
*Allium cepa*																■						■												
*Allium sativum*				■	■	■				■	■	■	■	■	■																			*
*Althaea officinalis*																						□	□	□										
*Anagallis foemina*				■																		■												
*Arctium minus*																																		
*Arctostaphylos uva-ursi*																																		
*Asparagus officinalis*	■																																	
*Calendula officinalis*																												■	■	■				
*Capsella bursa-pastoris*																																		
*Centaurium erythraea *ssp. *erythraea*	■	■																																
Centaurium erythraea ssp. majus majus							■	■																										
*Chelidonum majus*				■	■														■	■					■	■								
*Cichorium intybus*				■																														
*Crataegus monogyna*																																		
*Equisetum arvense*	■												■																					
*Eucalyptus globulus*																																		
*Fragaria vesca*																																		
*Hedera helix*																												■	■					
*Hypericum perforatum*				■	■	■				■	■	■	■	■	■													■	■	■				
*Juglans regia*							■			■																		■						*
*Lavandula angustifolia*																																		
*Malva sylvestris*	■			■																		■												*
*Marrubium vulgare*																						■												
*Melissa officinalis*													■	■	■																			
*Mentha spicata*																						■												
*Nerium oleander*																																		
*Ocimum basilicum*																																		
*Origanum vulgare*																																		
*Pinus sylvestris*				■																														
*Plantago lanceolata (root, leaf)*				■	■																	□ ■												
*Plantago major*				■																														
*Prunus spinosa*	■									■																								
*Quercus sp*.																						■												
*Rorippa nasturtium-aquaticum*	■																																	
*Rosa canina*										■,												■						,■						
*Rosmarinus officinalis*																			■	■		■	■								■	■		
*Rubus ulmifolius*																																		
*Ruscus aculeatus*																															■			
*Salvia officinalis*							■	■														■	■											
Sambucus nigra				■		■				■		■				■		■										■		■				
*Symphytum officinale ssp. officinale*																																		
*Tanacetum parthenium*								■	■		■	■																	■	■				
*Taraxacum officinale*	■	■		■	■																													
*Thymus vulgaris*	■	■								■																					■	■		
*Tilia platyphyllos*																			■															
*Tussilago farfara*				■a																														
*Urtica dioica*	■	■														■	■								■	■		■	■					
*Viola riviniana*																																		
*Viscum album*	■																																	

**a: **1: Cardiovascular system and haematology; 2: Dermatology; 3: Endocrinology, reproductive system liver and gall bladder; 4: Gastrointestinal tract; 5: Joints and rheumatism; 6: Infectious diseases, fever, anti-inflammatory and inmunostimulant; 7: Neurology and psychiatry; 8: Respiratory tract; 9: Urinary tract; 10: Ear, nose, throat, mouth and ophthalmology; 11: Other traditional applications. 12: Veterinary uses.

**b: **E: Commission E; S: ESCOP; W: WHO (): Positive Monograph; () negative Monograph; (■): It exists a Monograph but for other therapeutic category; (□): It exists Monograph for this therapeutic application, but the organ floral does not coincide.

* For veterinary use there is not Monograph.

Of the 92 species in the ethnobotanical catalogue, 39 do not appear in the published monographs, which indicates that their safety and efficacy are not officially recognized because of a lack of scientific study, despite the fact that several bioactive compounds and active ingredients of some plants have been recognized by a number of authors, as outlined below. A third of all plants belongs to the category related to dermatological disorders, the most frequently cited category in the Navarre Pyrenees. The extent to which the Navarre Pyrenees ethnopharmacology has been officially reviewed is 57,6% of the total, which suggests a large scope of research still to be done.

### Comments on some relevant species

The traditional ointment of the second bark of *Sambucus nigra *is barely mentioned in the scientific bibliography, could be a possible route for further pharmacological study because preliminary studies on the elder bark [84] have confirmed the presence of non-toxic ribosome-inactivating protein (RIP), leading to the inhibition of protein synthesis. According to Uncini-Manganelli *et al*. [85] and Girbes *et al*. [84] conjugation of RIP to monoclonal antibodies is a promising tool for cancer *therapy*. So a far-reaching study of this plant to test these biological properties may be of significant interest.

On the other hand, attention should be focused on the use of plants known popularly as "manzanilla" (chamomile), which correspond to the following species: *Chamaemelum nobile, Santolina chamaecyparissus *ssp. *squarrosa *and *Tanacetum parthenium*, generally used for gastrointestinal disorders. The difference which was observed between these three taxa is that from valley to valley the taxon known as "manzanilla" varies. Thus *Chamaemelum nobile *is used in the most western part of the region (which has the most humid climate), *Santolina chamaecyparissus *in the most Eastern parts (bordering the Central Pyrenees Region, a more Mediterranean climate), and *Tanacetum parthenium *only cultivated in kitchen gardens in the several villages of the most eastern valleys. This factor can be explained by the influence of the climate on the selection of plants named and used as chamomiles. These similar species, and with the same use, may be used interchangeably, depending on their accessibility and availability, among other reasons [86].

*Hypericum perforatum *is cited as an excellent plant for the treatment of diarrhoea. It is more commonly used for this gastrointestinal problem than for other purposes such as, for example, as a tranquillizer, as happens frequently in other cultures of the world. This plant was cited along with *Verbena officinalis *as a tranquillizer; taken during autumn and winter to raise the spirits because of the depression or physical decline which some inhabitants of this region suffered during these period of the year. Moreover, this plant is usually harvested on St. John's Eve along with *Sambucus nigra*, *Verbena officinalis *and *Rosa canina*, among others; this harvest is regarded as an ancestral custom, and the magical or medicinal properties of these plants are believed to increase that night.

*Verbena officinalis *is also used in folk medicine as an expectorant and anti-rheumatic and anti-inflammatory substance. Although some of these uses have been proven scientifically [87], WHO, ESCOP and the E Commission have not published any monographs that ensure their safe and effective application.

Regarding the mistletoe (*Viscum album*), all the informants agreed that the only plant which "worked" was the one which appears on species such as blackthorn (*Prunus spinosa*), apple (*Malus sylvestris*) or other species in the rose family. *Viscum album*, is characterized by its action on the cardiovascular system. Nevertheless, this is the sub-species *album *[65], which is a parasite on plants of the rose family, and so it would seem that the active compounds could vary according to the particular sub-species of *Viscum album *in question.

*Scrophularia auriculata *is a species barely mentioned in terms of traditional medicine in scientific bibliography. However, studies revealing glycoterpenoids in its chemical composition have been carried out [88]. These compounds reduce inflammatory injury and suppress cellular infiltration, which would corroborate the traditional application of this plant, although it has not yet been officially approved, as is the case also with *Dorycnium pentaphyllum *and *Saxifraga longifolia*, which have not been the object of no critical evaluation of their scientific properties; and therefore, no monographs have been published and this study marks the first time they have been recorded in the context of medicinal purposes.

*Rosa *sp., *Viola *sp., *Ocimum basilicum, Origanum vulgare *and *Nerium oleander *are the only species about which negative monograph have been published (71), which advise against prescribing the fruit on the grounds that its therapeutic effects have not been proven sufficiently. Another plant about which the E Commission has reported negatively is *Fragaria vesca *which, although scarce in the zone, it is used to relieve 'prostate discomfort'. Its popular use as a diuretic and antigout treatment has been recorded in other areas, using leaves and fruits respectively [89,90], and the beneficial effect of this species (among others) has been scientifically proven for the post-operatory period in prostatic adenomectomy [91].

In this context, therefore, more detailed, pharmacological study of these and other interesting species may be of considerable value, not only to validate their use and the future validation of traditional medicine in rural areas, but also, as recommended by WHO [92], because they may provide support for sustainable development projects in these areas, as mentioned by Pieroni *et al*. [81,93].

## 4. Conclusion

This ethnobotanical and medical study carried out in the Navarre Pyrenees region provides examples of several interesting medicinal plant uses worthy of pharmacological research, as is the case of *Sambucus nigra*, *Fragaria vesca *and *Scrophularia nodosa*.

At the same time, as well as the need for an exact floral catalogue of the zone to provide more accurate results in the Ethnobotany index, the study reflects a decline in ethnopharmacological knowledge, given the results of the quantitative analyses in the above-mentioned index, Shannon-Wiener, FIC, and the high number of species mentioned by less than three informants, in comparison to other published studies of Pyrenean regions.

Apart from quantitative data, there are differences about the consume of edible plants, owing to the fast that Navarre Pyrenees is more similar to other Temperate climate areas in this aspect than to the rest of Pyrenees regions (with Mediterranean climate). In addition, it is well distinguished edible plants and medicinal plants. Spirits, despite its social use, are drinked with digestive purposes and therefore the may be consider as medicinals. Folk costumes and beliefs are also manteined in Navarra nowadays as for instance the use of plants in order to protect people from diseases and evil spirits.

A wide-ranging official review of all non-published drugs by the institutions responsible for drug safety and efficacy, such as WHO, ESCOP and the E Commission of the German Department of Health is necessary, because, apart from fostering the use of more medicinal plants, this may foment the development of sustainable development plans in rural areas, such as the Navarre Pyrenees, where the loss of ethnobotanical and medical culture is already in evidence.

## Authors' contributions

SA conceived of the study, carried out the field work, the quantitative ethnobotanical analysis and the revision on monographs; and drafted the manuscript. RYC conceived of the study and participated in its design and coordination and participated in the field work and writing the manuscript. MIC conceived of the study and participated in its design and coordination and participated in the field work and in the revision on monographs.

## Supplementary Material

Additional file 1 Medicinal Plants used in the Navarre Pyrenees. Click here for file
