# Supplementary material for: First comprehensive contribution to medical ethnobotany of Western Pyrenees
Source: J Ethnobiol Ethnomed. 2007 Jun 6;3:26. doi: 10.1186/1746-4269-3-26 (PMC1904192; doi:10.1186/1746-4269-3-26)
Supplement: Additional file 1 — Medicinal Plants used in the Navarre Pyrenees. [file 1746-4269-3-26-S1.doc]

Additional file 1: Medicinal plants used in the Navarre Pyrenees area.

| **Nº** | Species | Local name(spanish, euskera) | PU ^a^ | Preparation ^b^ | Popular use (medical category, citations number for this application) | CF |
| --- | --- | --- | --- | --- | --- | --- |
|  | **Apocynaceae** |  |  |  |  |  |
| 1 | *Nerium oleander* L. (PAMP 18785) | Adelfa | L | FU | Sinusitis (**10**, 1) | 1 |
|  | **Araliaceae** |  |  |  |  |  |
| 2 | *Hedera helix* L. (PAMP 18786) | Hiedra | LS | M | Toothache (**10**, 1) | 1 |
|  | **Asteraceae** |  |  |  |  |  |
| 3 | *Achillea millefolium* L.  (PAMP 18636-18638) | Milenrama | F | I  O | Period pains (**3**, 1); digestive (**4**, 1)  Spots (**2**, 1) | 3 |
| 4 | *Arctium minus* Bernh. (PAMP 18639) | Bardana, hojas de lamparagón | R, L | MAL, P | Wounds, spots (**2**, 4) | 4 |
| 5 | *Bellis perennis* L. (PAMP 19879) | Margarita | FT | R | For health protection **(11, 1)** | 1 |
| 6 | *Calendula officinalis* L*.* (PAMP 18787, 18788) | Caléndula | F | O, OW  MAO | Burns, wounds (**2**, 2)  Earache (**10**, 1) | 3 |
| 7 | *Carlina acaulis* L*.* (PAMP 19880) | *Eguzkilore* | AP | R | Protection from diseases and evil spirits (**11**, 2) | 2 |
| 8 | *Chamaemelum nobile* (L.) All. (PAMP 18640, 18641) | Manzanilla, manzanilla del monte, *kamamila* | F | I, MAN  OW  MAO | Intestinal pain, constipation and stomach ache (**4**, 18); sedative (**7**,1)  To clean the eyes (**10**, 1)  Wounds (**2**, 1) | 21 |
| 9 | *Cichorium intybus* L. (PAMP 18789) | Achicoria silvestre | LX | DA | Warts (**2**, 1) | 1 |
| 10 | *Jasonia glutinosa* (L.) DC. (PAMP 18642) | Té de roca, té de peña | F | I | Stimulating (**7**, 9); digestive (**4**, 4) | 13 |
| **Nº** | Species | Local name(spanish, euskera) | PU ^a^ | Preparation ^b^ | Popular use (medical category, citations number for this application) | CF |
| 11 | *Jasonia tuberosa* (L.) DC. (PAMP 19881) | Té de suelo | AP | I | Stimulating (**7**, 1) | 1 |
| 12 | *Lactuca serriola* L. (PAMP 19882) | Lechecino | LX | DA | Warts (**2**, 1) | 1 |
| 13 | *Santolina chamaecyparissus* L. ssp*. squarrosa* (DC) Nyman (PAMP 18643-18649, 18749) | Manzanilla, manzanilla del monte | F | I, MAN  SB  O | Digestive (**4**, 25)  Women’s intimate hygiene (**3**, 1)  Wounds (**2**, 1) | 27 |
| 14 | *Sonchus oleraceus* L. (PAMP 19883) | Lechecino | LX | DA | Warts (**2**, 1) | 1 |
| 15 | *Tanacetum parthenium* Schultz Bip. (PAMP 18650) | Manzanilla, manzanilla de la huerta | F | I  OW  SB | Digestive (**4**, 2)  To clean the eyes (**10**, 1)  Women’s intimate hygiene (**3**, 1) | 4 |
| 16 | *Taraxacum officinale* group*.* (PAMP 18651) | Diente de león | R, LX | MAO  DA  C | Spring cure for the liver (**3**, 1)  Warts (**2**, 1)  To purify the body (**1**, 1) | 3 |
| 17 | *Tussilago farfara* L. (PAMP 18652-18656) | Patamula | L | P, DA  I | Boils and wounds (**2**, 5)  Cold (**8**, 1) | 6 |
|  | **Betulaceae** |  |  |  |  |  |
| 18 | *Corylus avellana* *L.* (PAMP 18790) | Avellano | L | MAO | Skin diseases (**2**, 1) | 1 |
|  | **Boraginaceae** |  |  |  |  |  |
| 19 | *Symphytum officinale* L*.* (PAMP 18657, 18658) | Consuelda | R | O | Anti-inflammatory (**6**, 1); blows, rheumatism, osteoarthritis (**5**, 1) | 2 |
| **Nº** | Species | Local name(spanish, euskera) | PU ^a^ | Preparation ^b^ | Popular use (medical category, citations number for this application) | CF |
| 20 | *Symphytum tuberosum* L*.* (PAMP 18659) | Consuelda menor | R | P | Anti-inflammatory (**6**, 2) | 2 |
|  | **Brassicaceae** |  |  |  |  |  |
| 21 | *Brassica oleracea* L. var*. oleracea* (PAMP 19884) | Berza | L | O | Herpes (**2**, 1) | 1 |
| 22 | *Capsella bursa-pastoris* (L.) Medik (PAMP 18807) | Bolsa de pastor | AP | I | To regulate menstruation (**3**, 1) | 1 |
| 23 | *Rorippa nasturtium-aquaticum* (L.) Hayek (PAMP 18791) | Berro | L | C | Blood problems (e.g. blood-flow) (**1**, 1) | 1 |
|  | **Buxaceae** |  |  |  |  |  |
| 24 | *Buxus sempervirens* L. (PAMP 18660) | Boj | L | R | Warts (**2**, 1) | 1 |
|  | **Caprifoliaceae** |  |  |  |  |  |
| 25 | *Sambucus nigra* L. (PAMP 18661-18668) | Saúco, *sabuko* | F, L, FR  B | I, IH, D, SY, C,  MAN, FU, P  O  OW | Cold, and headaches caused by a cold (**8**, 17); stomach ache (**4**, 1)  Toothache and sore throat, (**10**, 16); anti-inflammatory, pains (**6**, 2);  abscess, burns, spots, wounds and psoriasis (**2**, 14)  Problems with the eyes (swelling…) (**10**, 1) | 51 |
|  | **Crassulaceae** |  |  |  |  |  |
| 26 | *Hylotelephium maximum* (L.) Holub (PAMP 18669, 18746) | Curamal, curamales, curalotodo | L | DA | Spots, pricks, wounds and to clean gangrene (**2**, 9) | 9 |
| 27 | *Sedum* sp. (PAMP 19885) | Hierba para almorranas | AP | P | Piles (**2**, 2) | 2 |
| 28 | *Umbilicus rupestris* (Salisb.) Dandy (PAMP 18792) | Ombligo de venus | L | P | Injuries (**2**, 1) | 1 |
|  | **Equisetaceae** |  |  |  |  |  |
| **Nº** | Species | Local name(spanish, euskera) | PU ^a^ | Preparation ^b^ | Popular use (medical category, citations number for this application) | CF |
| 29 | *Equisetum arvense* L. (PAMP 18670, 18671) | *Axari buzten*, cola de caballo | SP | I, D  P | Hypertension (**1**, 1); calcium deficiency (**5**, 2); diuretic (**9**, 2); kidney stones (**3**, 1)  Sore throat (**10**, 1) | 7 |
|  | **Ericaceae** |  |  |  |  |  |
| 30 | *Arctostaphylos uva-ursi* (L.) Sprengel (PAMP 18793) | Gayuba | S, L | I | Kidney stones (**9**, 1); prostate problems (**3**, 1) | 2 |
|  | **Fabaceae** |  |  |  |  |  |
| 31 | *Dorycnium pentaphyllum* Scop*.* (PAMP 18672) | Planta para las piedras del riñón | AP | I | Kidney stones (**9**, 1) | 1 |
|  | **Fagaceae** |  |  |  |  |  |
| 32 | *Fagus sylvatica* L. ssp*. sylvatica* (PAMP 18794) | Haya, *pagoa* | B, L | B | Cold (**8**, 1); deep wounds caused by an axe **(2,** 1) | 2 |
| 33 | *Quercus* sp. (18806) | Roble | B | B | Cold (**8**, 1) | 1 |
|  | **Gentianaceae** |  |  |  |  |  |
| 34 | *Centaurium erythraea* Rafn (PAMP 18673) |  | AP | I | To purify the blood (**1**, 2) | 2 |
| 35 | *Centaurium erythraea* Rafn ssp*. majus* (Hoffmanns. & Link) M. Laínz (PAMP 18674) | Hierba para el hígado | AP | I | Liver pain (**3**, 1); sedative (**7**, 1) | 2 |
|  | **Geraniaceae** |  |  |  |  |  |
| 36 | *Geranium robertianum* L. (PAMP 18675) | - | AP | P | Wounds (**2**, 1) | 1 |
| **Nº** | Species | Local name(spanish, euskera) | PU ^a^ | Preparation ^b^ | Popular use (medical category, citations number for this application) | CF |
|  | **Guttiferae** |  |  |  |  |  |
| 37 | *Hypericum perforatum* L. (PAMP 18676-18680) | Hipérico, hipérico del roncal, hierba de San Juan | FT | I  MAO  MAL | Stimulanting (**7**, 1); diarrhoea (**4**, 6)  Burns, wounds, skin troubles (**2**, 5); sore throat (**10**, 1)  Muscular pains (**5**, 2) | 15 |
|  | **Juglandaceae** |  |  |  |  |  |
| 38 | *Juglans regia* L. (PAMP 18681-18683) | Nogal | BD, L,  FR | D, MAN  G  MAW | Stomach ache, diarrhoea caused by stomach colds (**4**, 3); to heal wounds in animals (**12**, 3)  To clean the mouth (**10**, 1)  For menstruation problems (**3**, 1) | 8 |
|  | **Lamiaceae** |  |  |  |  |  |
| 39 | *Lavandula angustifolia* Bub. ssp. *pyrenaica* (DC) Guinea (PAMP 18684, 18685) | Lavanda, espliego | F | I  PE | Digestive (**4**, 2)  Other tradicional uses (for foot odour) (**11**, 1) | 3 |
| 40 | *Marrubium vulgare* L. (PAMP 18686) | Marrubio blanco | AP | I | Old colds (**8**, 1) | 1 |
| 41 | *Melissa officinalis* L. (PAMP 18687-18690) | Melisa, salvia | AP, L | I  P | Digestive (**4**, 1); sedative (**7**, 1)  Problems with tendons and joints, rheumatism (**5**, 1) | 3 |
| 42 | *Mentha longifolia* Huds. (PAMP 18691) | Menta | AP | I | Stomach ache (**4**, 1) | 1 |
| 43 | *Mentha spicata* L*.* (PAMP 18692-18694) | Menta | L | I | Digestive (**4**, 4) | 4 |
| 44 | *Mentha suaveolens* Ehrh. (PAMP 18803) | *Menda*, menta | L | I | Digestive (**4**, 1); sedative (**7**, 1) | 2 |
| 45 | *Ocimum basilicum* L*.* (PAMP 18695) | Hierbabuena | L | I | Digestive (**4**, 1) | 1 |
| **Nº** | Species | Local name(spanish, euskera) | PU ^a^ | Preparation ^b^ | Popular use (medical category, citations number for this application) | CF |
| 46 | *Origanum vulgare* L. (PAMP 18695) | Orégano | F | I | Digestive (**4**, 1) | 1 |
| 47 | *Rosmarinus officinalis* L*.* (PAMP 18804) | Romero | AP | I  MAL, MAW  PE | To prevent and cure colds (**8**, 1); to clean blood (**1**, 1); digestive (**4**, 1); stimulant (**7**, 1)  Muscular pain and rheumatism (**5**, 1)  For foot odour (**11**, 1) | 6 |
| 48 | *Salvia officinalis* L. (PAMP 18696-18697) | Salvia | AP | I | Cold (**8**, 1); for menstrual pain (**3**, 1) | 2 |
| 49 | *Sideritis hyssopifolia* L*.* ssp. *guillonii* (Timb.-Lagr.) Rouy (PAMP 18698, 18699, 18750, 18751) | Té de roca | AP | I | Stomach ache, digestive (**4**, 5) | 5 |
| 50 | *Thymus praecox* Opiz subsp*. polytrichus* (A. Kerner ex Barbás) Jalas (PAMP 18700) | Orégano de monte | AP | I | Cold (**8**, 1) | 1 |
| 51 | *Thymus vulgaris* L*.* ssp*. vulgaris* (PAMP 18701-18703) | Tomillo | AP | I  PE  G  MAO | To regulate blood pressure (**1**, 1); to prevent and cure colds (**8**, 4); for stomach colds, stomach ache (**4**, 2)  For foot odour (**11**, 1)  To clean the mouth (**10**, 1)  Deafness caused by wax plugs (**10**, 2) | 11 |
|  | **Liliaceae** |  |  |  |  |  |
| 52 | *Allium ampeloprasum* L. (PAMP 19886) | Puerro | S, L | D | To purify blood (**1**, 1); diuretic (**9**, 1) | 2 |
| 53 | *Allium cepa* L. (PAMP 19887) | Cebolla | L | P, DA,  R | Infected spots, to heal (**2**, 14); fever (**6**, 1)  Cough (**8**, 1) | 16 |
| 54 | *Allium sativum* L*.* (PAMP 19888) | Ajo | L | I, MAN, P  R | Tapeworm (**4**, 1); rheumatism (**5**, 2); wounds, burn (**2**, 3); cold (**8**,1)  For distemper in dogs and cats (**12**, 1) | 8 |
| **Nº** | Species | Local name(spanish, euskera) | PU ^a^ | Preparation ^b^ | Popular use (medical category, citations number for this application) | CF |
| 55 | *Asparagus officinalis* L*.* (PAMP 19889) | Espárrago | S | C | To clean blood (**1**, 1) | 1 |
| 56 | *Ruscus aculeatus* L*.* (PAMP 18704, 18705) | Ispelco | AP | R | Protection from diseases and evil spirits (**11**, 3) | 3 |
|  | **Loranthaceae** |  |  |  |  |  |
| 57 | *Viscum album* L. (PAMP 18811) | *Bizko, migula, mihura***,** muérdago, muérdago de espino | S, L | I, D | To regulate blood pressure (**1**, 8) | 8 |
|  | **Lycoperdaceae** |  |  |  |  |  |
| 58 | *Lycoperdon* sp. (PAMP 19890) | Pedo de lobo | SR | P | Wounds (**2**, 2) | 2 |
|  | **Lythraceae** |  |  |  |  |  |
| 59 | *Lythrum salicaria* L*.* (PAMP 18706, 18707) | Hierba para las diarreas | FT | D | Diarrhoea (**4**, 1) | 1 |
|  | **Malvaceae** |  |  |  |  |  |
| 60 | *Althaea officinalis* L. (PAMP 18805) | Malvavisco | F | I | Cold (**8**, 1) | 1 |
| 61 | *Malva neglecta* Wallr (PAMP 18708) | Malva | AP | P | Wound (**2**, 1) | 1 |
| 62 | *Malva sylvestris* L.(PAMP 18709, 18710, 18747, 18748) | Malva | WP | I, D  P, O | To clean blood (**1**, 1); colds (**8**, 1)  For horses’ wounds (**12**, 4); boils (**2**, 3) | 9 |
|  | **Moraceae** |  |  |  |  |  |
| 63 | *Ficus carica* L. (PAMP 18801) | Higuera | LX  L | DA  FU | Warts (**2**, 3)  Gumboils (**10**, 1) | 4 |
|  | **Myrtaceae** |  |  |  |  |  |
| **Nº** | Species | Local name(spanish, euskera) | PU ^a^ | Preparation ^b^ | Popular use (medical category, citations number for this application) | CF |
| 64 | *Eucalyptus globulus* Labill (PAMP 18800) | Eucalipto | L | SY | Cold (**8**, 1) | 1 |
|  | **Papaveraceae** |  |  |  |  |  |
| 65 | *Chelidonium majus* L*.* (PAMP 1871, 18712) | Celidonia, *iodobelarra* | LX, L  S | DA, O  I | Wounds, fungal growth and warts (**2**, 5)  Sedative (**7**, 1); diuretic (**9**, 1) | 7 |
|  | **Pinaceae** |  |  |  |  |  |
| 66 | *Pinus sylvestris* L. (PAMP 18798) | Pino | B | P | Wounds (**2**, 1) | 1 |
|  | **Plantaginaceae** |  |  |  |  |  |
| 67 | *Plantago lanceolata* L*.* (PAMP 18713-18715) | Aquinácea, llantén, plantago menor | RZ, L | D  P, DA | Bronchitis (**8**, 2)  Mouth pain (**10**, 1); mouthache and bleeding gums (**2**, 1) | 4 |
| 68 | *Plantago major* L. (PAMP 18716, 18717) | Plantago mayor | L | P  DA | Mouthache and bleeding gums (**10**, 1)  Wounds (**2**, 4) | 5 |
|  | **Poaceae** |  |  |  |  |  |
| 69 | *Triticum aestivum* L*.* (18797) | Trigo | FR | P | Bronchial problems (**8**, 1) | 1 |
| 70 | *Zea mays* L. (PAMP 19891) | Maíz | F | I | Diuretic (**9**, 1) | 1 |
|  | **Primulaceae** |  |  |  |  |  |
| 71 | *Anagallis foemina* Mill. (PAMP 18718) | *Pasmobelarra* | AP | I  O | Respiratory infections (**8**, 1)  Spots (**2**, 1) | 2 |
|  | **Rhamnaceae** |  |  |  |  |  |
| **Nº** | **Species** | **Local name**  **(spanish, *euskera*)** | **PU ^a^** | **Preparation ^b^** | **Popular use (medical category, citations number for this application)** | **CF** |
| 72 | *Rhamnus alaternus* L*.* (PAMP 18796) | Carrasquilla, *karraskila* | BR | D | To make blood more fluid (**1**, 3); rheumatism (**5**, 1); cold (**8**, 1) | 5 |
|  | **Rosaceae** |  |  |  |  |  |
| 73 | *Agrimonia eupatoria* L*.* (PAMP 18719, 18720) | Agrimonia | AP | I, G | Sore throat (**10**, 2); stomach ache (**4**, 1); cold (**8**, 1) | 4 |
| 74 | *Crataegus monogyna* Jacq (PAMP 18721-18723) | Espino blanco, flor del corazón, gurrillón | F, FR | I, MAN, D | For problems in heart functioning, hypertension, varicose veins (**1**, 6) | 6 |
| 75 | *Fragaria vesca* L. ssp. *vesca* (PAMP 18802) | Fresa | R | D | Prostate problems (**3**, 1) | 1 |
| 76 | *Malus sylvestris* Mill. (PAMP 19892) | Manzano, *patxaka* | FR | MAN | Stomach ache (**4**, 1) | 1 |
| 77 | *Potentilla reptans* L. (PAMP 18795) | Cincoenrama | WP | P | Wounds (**2**, 1) | 1 |
| 78 | *Prunus spinosa* L. (PAMP 19893) | *Patxaran* | FR  BR, R | MAN  D | Stomach ache, diarrhoea caused by stomach colds (**4**, 9)  Hypertension, to make blood more fluid (**1**, 2) | 11 |
| 79 | *Rosa canina.* L. (PAMP 18725-18728, 18809) | Cabardas, cabarderas, rosa, escaramujo | FR  F, B | I, C, MAN  D, OW | Diarrhoea (**4**, 5); sore throat (**10**, 1); cold (**8**, 4)  Hypertension (**1**, 1); to clean the eyes (**10**, 3) | 1P |
| 80 | *Rubus ulmifolius* Schott (PAMP 18729) | Zarza, zarzamora | NS | I  G | Diarrhoea caused by stomach colds (**4**, 3)  For cleanness the mouth (**10**, 1) | 4 |
|  | **Rutaceae** |  |  |  |  |  |
| 81 | *Citrus aurantium* L*.* (PAMP 19894) | Naranjo | F | D | Prostate problems (**3**, 1) | 1 |
| 82 | *Citrus limon* (L.) Burm (PAMP 19895) | Limón | FR | J | Sore throat (**10**, 1) | 1 |
|  | **Saxifragaceae** |  |  |  |  |  |
| 83 | *Saxifraga longifolia* Lapeyr (PAMP 19896) |  | L | T | For deafness caused by wax plugs (**10**, 1) | 1 |
| **Nº** | **Species** | **Local name**  **(spanish, *euskera*)** | **PU ^a^** | **Preparation ^b^** | **Popular use (medical category, citations number for this application)** | **CF** |
|  | **Scrophulariaceae** |  |  |  |  |  |
| 84 | *Scrophularia auriculata* L. (PAMP 18730) | Escrofularia | WP | P | Antibiotic and anti-inflammatory (**6**, 1) | 1 |
| 85 | *Verbascum thapsus* L. (PAMP 18731-18733) | Hierba para almorranas | R, L | R  I | Hemorrhoids (**2**, 1)  Respiratory infections (**8**, 2) | 3 |
|  | **Solanaceae** |  |  |  |  |  |
| 86 | *Lycopersicum esculentum* Mill. (PAMP 19897) | Tomate | F | P, DA | Toothache and sore throat (**10**, 2) | 2 |
|  | **Tamaricaceae** |  |  |  |  |  |
| 87 | *Tamarix gallica* L. (PAMP 18810) | Taray |  | P | For foot odour (**11**, 1) | 1 |
|  | **Tiliaceae** |  |  |  |  |  |
| 88 | *Tilia platyphyllos* Scop. (PAMP 18734-18738, 18752) | Tilo, tilo de montaña | FT | I | Sedative (**7**, 13); for sick animals (**12**, 1) | 14 |
|  | **Umbelliferae** |  |  |  |  |  |
| 89 | *Foeniculum vulgare* L. (PAMP 19898) | Hinojo | AP | R | Other traditional uses (protection from diseases and evil spirits) (**11**, 1) | 1 |
|  | **Urticaceae** |  |  |  |  |  |
| 90 | *Urtica dioica* L. (PAMP 18739) | Ortiga, *atsunes* | AP | C, I  F | To improve blood circulation, cholesterol (**1**, 9); for weak hair (**10**, 2); depurative, diuretic (**9**, 2);  Backache and rheumatism (**5**, 7); pains (**6**, 2) | 22 |
| **Nº** | **Species** | **Local name**  **(spanish, *euskera*)** | **PU ^a^** | **Preparation ^b^** | **Popular use (medical category, citations number for this application)** | **CF** |
|  | **Verbenaceae** |  |  |  |  |  |
| 91 | *Verbena officinalis* L. (PAMP 18740-18745) | Verbena | AP | P  I | Sore throat (**10**, 2); cold, chest congestion, bronchitis, asthma and bronchitis (**8**, 9); wounds and to ripen the grains (**2**, 5); pains, anti-inflammatory (**6**, 1)  Other traditional uses (for old diseases) (**11**, 1); stimulanting (**7**, 1) | 19 |
|  | **Violaceae** |  |  |  |  |  |
| 92 | *Viola riviniana* Reichenb. (PAMP 18808) | Violeta | F | I | Sinusitis (**10**, 1) | 1 |

**^a^ Parts used:** AP: aerial part; B: bark; BD: bud; BR: branch; F: flower; FR: fruits; FT: floral top; L: leaf; LS: leaf-stalk; LX: latex; NS: new shoot; R: root; RZ: rhizome; S: stem; SP: sterile plant; SR: spore; WP: whole plant.

**^b^ Preparation.** B: bath; C: comestible; DA: direct application; D: decoction; F: friction; FU: fume; G: gargling; I: infusion; IH: inhalation; J: juice; MAL: maceration in alcohol; MAN: maceration in anisette; MAO: maceration in oil; MAW: maceration in wine; M: to masticate; O: ointment; OW: ocular washed; PE: pediluvium; P: poultice; R: rite; SB: sitz bath; SY: syrup.

**Therapeutic category**. 1: Cardiovascular system and haematology; 2: Dermatology; 3: Endocrinology, reproductive system liver and gall bladder; 4: Gastrointestinal tract; 5: Joints and rheumatism; 6: Infectious diseases, fever, anti-inflammatory and inmunostimulanting; 7: Neurology and psychiatry; 8: Respiratory tract; 9: Urinary tract; 10: Ear, nose, throat, mouth and ophthalmology; 11: Other traditional applications; 12: Veterinary uses.
